# Fabrication of Novel g-C_3_N_4_@Bi/Bi_2_O_2_CO_3_ Z-Scheme Heterojunction with Meliorated Light Absorption and Efficient Charge Separation for Superior Photocatalytic Performance

**DOI:** 10.3390/molecules27238336

**Published:** 2022-11-29

**Authors:** Hongxia Fan, Xiaohui Ma, Xinyang Li, Li Yang, Yongzhong Bian, Wenjun Li

**Affiliations:** Beijing Key Laboratory for Science and Application of Functional Molecular and Crystalline Materials, University of Science and Technology Beijing, Beijing 100083, China

**Keywords:** photocatalytic, Bi_2_O_2_CO_3_, g-C_3_N_4_, Z-scheme heterojunction

## Abstract

Herein, a novel g-C_3_N_4_@Bi/Bi_2_O_2_CO_3_ Z-scheme heterojunction was synthesized via simple methods. UV/Vis diffuse reflectance spectroscopy (DRS) revealed that the visible light absorption range of heterojunction composites was broadened from 400 nm to 500 nm compared to bare Bi_2_O_2_CO_3_. The XRD, XPS and TEM results demonstrated that metal Bi was introduced into g-C_3_N_4_@Bi/Bi_2_O_2_CO_3_ composites, and Bi may act as an electronic bridge in the heterojunction. Metal Bi elevated the separation efficiency of carriers, which was demonstrated by photocurrent and photoluminescence. The performance of samples was assessed via the degradation of Rhodamine B (RhB), and the results exhibited that g-C_3_N_4_@Bi/Bi_2_O_2_CO_3_ possessed notably boosted photocatalytic activity compared with g-C_3_N_4_, Bi_2_O_2_CO_3_ and other binary composites. The heterojunction photocatalysts possessed good photostability and recyclability in triplicate cycling tests. Radical trapping studies identified that h^+^ and •O_2_^−^ were two primary active species during the degradation reaction. Based on the energy band position and trapping radical experiments, the possible reaction mechanism of the indirect Z-scheme heterojunction was also proposed. This work could provide an effective reference to design and establish a heterojunction for improving the photocatalytic activity of Bi_2_O_2_CO_3_.

## 1. Introduction

With the increasingly serious problem of environmental pollution, people have been looking for available approaches to solve pollution challenges [1,2]. Advanced oxidation processes and photocatalysis in water treatment receive more and more attention because they could utilize solar energy to degrade organic pollutants [3,4]. Photocatalytic technology uses photocatalysts to produce active species (e.g., h^+^, •O_2_^−^, •OH) to oxidize organic pollutants in the photocatalysis process. Therefore, the development of efficient visible light photocatalysts is decisive for photocatalytic technology [5,6]. Recently, bismuth-based photocatalysts have attracted extensive attention, such as BiVO_4_ [7,8], Bi_2_O_2_CO_3_ [9,10], BiOX [11,12], and Bi_2_O_3_ [13,14]. Among them, Bi_2_O_2_CO_3_ (BOC) is deemed to be an attractive semiconductor for degrading organic dyes [15,16]. Deplorably, the poor activity of unmodified Bi_2_O_2_CO_3_ limits its environmental application because of the wide band gap. At present, many efforts have been attempted to improve the photocatalytic activity of Bi_2_O_2_CO_3_, such as by controlling morphology [17,18], constructing heterojunctions [19,20], and loading precious metals [21]. Constructing a heterojunction is considered as an ideal method because it can expand light absorption and improve carrier separation efficiency at the same time. Gao et al. [19] reported that the p-n BiOI/Bi_2_O_2_CO_3_ heterojunction presented splendid photocatalytic performance for MB and RhB. Huang et al. [22] synthesized a Bi_2_O_2_CO_3_/Bi_2_WO_6_ heterostructure, which possessed outstanding photocatalytic degradation activity for RhB. Even so, due to unsatisfactory activity, it is still necessary to explore other Bi_2_O_2_CO_3_-based photocatalysts for organic degradation.

More recently, g-C_3_N_4_ (CN) was applied for photocatalytic degradation because of its stability, excellent absorption properties, easy fabrication, and suitable band gap [23,24,25]. Based on the matched energy band levels of BOC and CN, they could form a direct Z-scheme heterojunction for elevated photodegradation activity. The direct Z-scheme heterojunctions are not sufficiently efficacious to boost carrier separation, resulting from the unsatisfactory e^−^ transport ability of BOC. It has been reported that the use of metal (Au and Ag, etc.) as Z-scheme bridges to form indirect Z-scheme heterojunctions could further accelerate the separation efficiency of photocarriers and thus promote the photocatalytic activity of a direct Z-scheme heterojunction system [26,27]. Owing to its opportune work function and outstanding electrical conductivity, low-cost metal bismuth (Bi) is expected to become a substitute for precious metals as an electronic bridge and gradually come into people’s view [28,29]. Additionally, a Bi bridge is acquired via the in situ reduction of BOC, which is awfully conducing to the constitution of Bi/BOC compact heterostructure. Thus, Bi as the bridge in the heterostructure g-C_3_N_4_@Bi/Bi_2_O_2_CO_3_ heterojunction is anticipated to remarkably promote charge separation for high activity. The photocatalytic degradation of the g-C_3_N_4_@Bi/Bi_2_O_2_CO_3_ heterojunction system has been not reported.

In this work, a novel g-C_3_N_4_@Bi/Bi_2_O_2_CO_3_ Z-scheme heterojunction was successfully constructed. Benefiting from the formed heterojunction, g-C_3_N_4_@Bi/Bi_2_O_2_CO_3_ composites possessed notably enhanced photocatalytic degradation activity of RhB due to promoted carriers separation efficiency and expanded photo-response range. We studied the g-C_3_N_4_@Bi/Bi_2_O_2_CO_3_ heterojunction system with an electron-conduction bridge under a photodegradation experiment.

## 2. Results and Discussion

**Figure 1:**
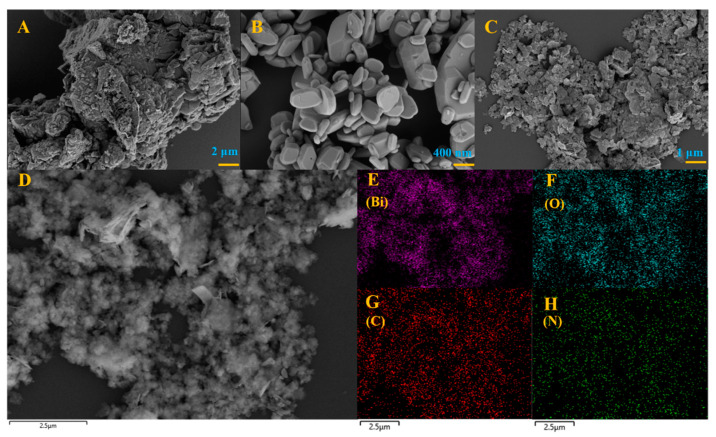
The SEM of BOC (**A**), CN (**B**) and BOC-CN-Bi (**C**,**D**), the mapping image of BOC-CN-Bi in (**E**) (Bi), (**F**) (O), (**G**) (C) and (**H**) (N).

**Figure 2:**
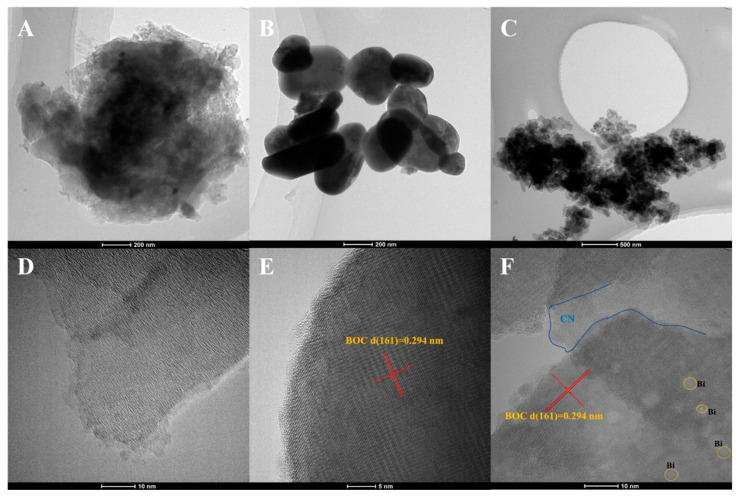
The TEM and HRTEM of CN (**A**,**D**), BOC (**B**,**E**) and BOC-CN-Bi (**C**,**F**).

The microscopic structure and morphology of BOC, CN and BOC-CN-Bi photocatalysts are analyzed via SEM and EDS. Pure BOC is an irregular agglomerated grain structure (Figure 1A). As shown in Figure 1B, g-C_3_N_4_ possesses a sheet structure that is tens of nanometers thick. The microstructure of BOC-CN-Bi heterojunction composites is shown in Figure 1C. Figure 1E–H displays SEM images and elemental mapping images of BOC-CN-Bi composites, which manifests an even distribution of Bi, C, O and N elements. Table S1 (Supplementary Materials) shows that the Bi element is excessive, indicating the presence of metallic Bi in BOC-CN-Bi composites. The SEM results reveal that BOC-CN-Bi Z-scheme heterojunction composites are successfully prepared. Figure 2A–F shows TEM and HRTEM images of CN, BOC, and BOC-CN-Bi samples. The morphology shown by TEM images was in good agreement with the SEM results. In Figure 2D, CN possesses an amorphous structure as reported in the literature. Furthermore, pure BOC has good crystallinity and its (161) crystal plane spacing is 0.294 nm (Figure 2E). Interestingly, for BOC-CN-Bi (Figure 2F), both CN and BOC could be clearly observed. Meanwhile, metal Bi can also be found by reduction with EG. These results definitely illustrate the successful preparation of BOC-CN-Bi ternary heterostructure composites.

**Figure 3:**
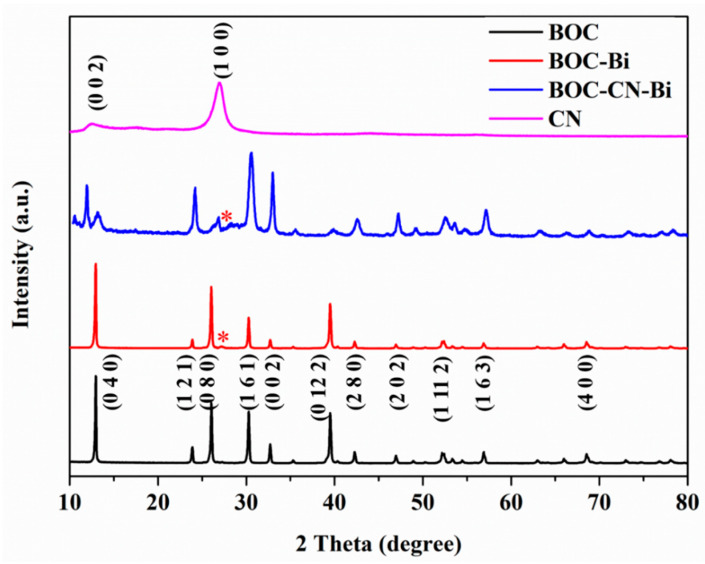
XRD patterns of BOC, CN, BOC-Bi and BOC-CN-Bi photocatalysts (* indicates peaks of metallic Bi).

Figure 3 displays the XRD patterns of as-prepared BOC, CN, BOC-Bi and BOC-CN-Bi photocatalysts. In the XRD patterns of BOC, BOC-Bi and BOC-CN-Bi composites, the peaks of BOC match with the orthorhombic phase of BOC (JCPDS card No. 84-1752). Eleven major peaks at 12.91°, 23.88°, 26.07°, 30.27°, 32.64°, 39.49°, 42.23°, 46.89°, 52.28°, 56.84° and 68.62° are indexed to the planes of (0 4 0), (1 2 1), (0 8 0), (1 6 1), (0 0 2), (0 12 2), (2 8 0), (2 0 2), (1 11 2), (1 6 3) and (4 0 0), respectively. Simultaneously, compared to BOC, there is a new weak peak at 27.16° in the patterns of BOC-Bi and BOC-CN-Bi composites, which can be indexed to metallic Bi [30]. The peaks ((0 0 2) and (1 0 0)) of CN are consistent with that reported in the literature [31]. The peaks of single BOC, CN and Bi could be observed in the patterns of BOC-CN-Bi composites. Additionally, no impurity peaks are detected in the XRD patterns. The aforementioned results indicate that BOC-CN-Bi Z-scheme heterojunction composites have been successfully prepared. The XRD results support SEM and EDS results and will be further determined by XPS analysis.

**Figure 4:**
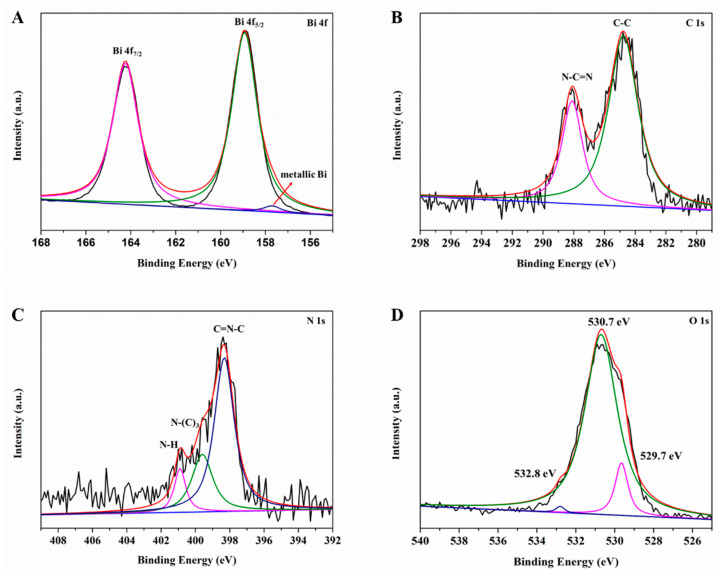
XPS spectra: (**A**) Bi 4f, (**B**) C 1s, (**C**) N 1s and (**D**) O 1s of BOC-CN-Bi composites.

The surface chemical compositions and the oxidation states of BOC-CN-Bi composites are analyzed via XPS, further confirming the coexistence of BOC, CN and Bi in heterojunction composites. The peaks at 164.3 and 158.9 eV correspond to Bi 4f_7/2_ and Bi 4f_5/2_, respectively (Figure 4A). The bismuth is a Bi^3+^ cation in the BOC-CN-Bi composite [32]. The peak located at 157.7 eV is attributed to metallic Bi in the BOC-CN-Bi composites. Figure 4B shows two typical peaks of C 1s situate at 288.1 and 284.8 eV, which associates with N-C=N and C-C, respectively. The N-C=N is caused by the sp^2^-bonded carbon in the nitrogenous aromatic ring. The C-C bond is caused by amorphous or graphitic carbons. In Figure 4C, the N 1s peak of composites is fitted as three peaks at 398.3, 399.6 and 400.9 eV, corresponding to sp2-hybridized nitrogen involved in triazine rings (C=N-C), tertiary N bonded in N-(C)_3_ groups, and N-H groups, respectively [33,34]. As shown in Figure 4D, the peaks at 529.7, 530.7, and 532.8 eV are assigned to Bi-O binding, carbonate ions, and absorbed H_2_O on the surface [35], respectively. Obviously, the aforementioned results confirm the formation of BOC-CN-Bi Z-scheme heterojunction.

**Figure 5:**
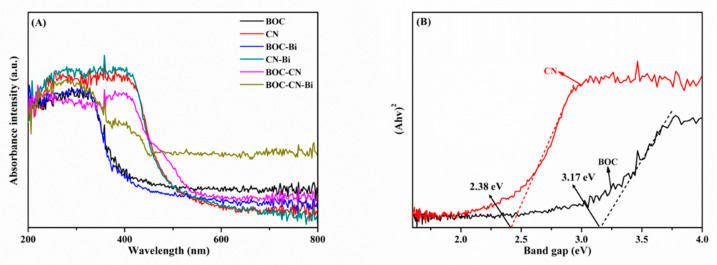
The DRS (**A**) of the as-obtained samples; the band gaps (**B**) of BOC and CN.

The UV-vis diffuse reflectance spectra are acquired to assess the optical response range of all samples. Pristine BOC and CN reveal severally conspicuous absorption edges at approximately 400 and 500 nm (Figure 5A). The absorption edges for BOC-Bi and CN-Bi possess a slight redshift compared to pristine BOC and CN, which suggests that metal Bi could slightly ameliorate the visible light absorption capacity of photocatalysts [36,37]. Intriguingly, BOC-CN-Bi ternary photocatalysts have a wider visible light absorption range than BOC and BOC-Bi, insinuating the likelihood of better photocatalytic performance for BOC-CN-Bi ternary photocatalysts. The wider visible light absorption range assures that BOC-CN-Bi could provoke ample photoinduced e^−^-h^+^ pairs. As presented in Figure 5B, the band gap of pristine BOC and CN are estimated as 3.17 and 2.38 eV via the Kubelka-Munk formula. Furthermore, the band gap of all composites is shown in Figure S1. The band gap for BOC-CN-Bi is 2.6 eV. Compared with BOC, the BOC-CN-Bi composite has a narrower band gap and higher carrier separation for efficient photocatalytic hydrogen production.

**Figure 6:**
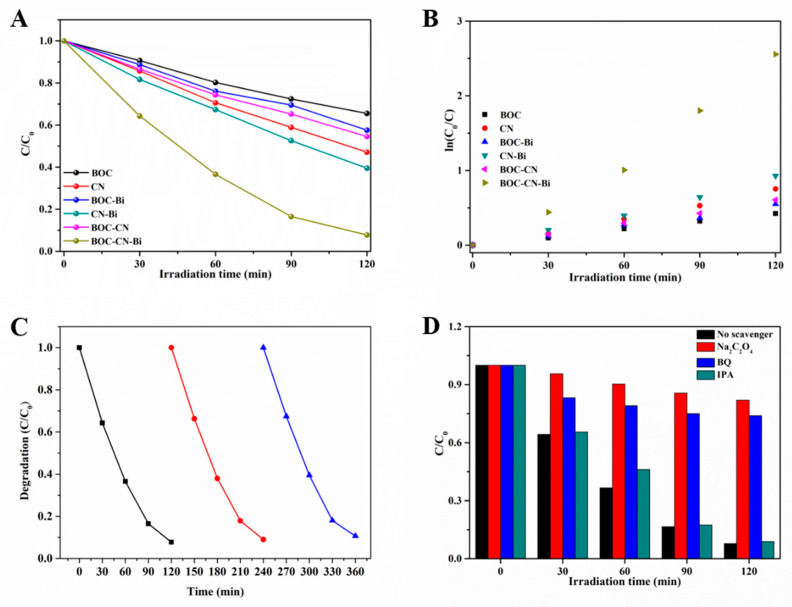
The photodegradation of different organic pollutants (**A**) and kinetics of the RhB decomposition (**B**) over as-prepared samples, the cycling experiments (**C**) and the trapping experiment (**D**) the result of BOC-CN-Bi ternary composites by degrading RhB.

In Figure 6A, the photocatalytic performance of all photocatalysts is evaluated via the photodegradation of the rhodamine B (RhB) under visible light (≥420 nm). Before the photoreaction process, all samples were kept in the dark to realize the adsorption-desorption equilibrium. The value of RhB adsorption for all samples is shown in Table S2 (Supplementary Materials). Pristine BOC reveals an inconsequential photodegradation rate of 30% after 120 min because of its wide band gap. As for CN, only 50% of RhB is disintegrated after 120 min. However, after the introduction of metal Bi, the binary BOC-Bi and CN-Bi exhibit better degradation rates (40% and 60%, respectively). Simultaneously, the photodegradation rate of the RhB for the BOC-CN compound is 45%. Interestingly, the ternary composites (BOC-CN-Bi) reveal the best photocatalytic activity compared to other samples, which is approximately 93% after 120 min. Additionally, the ternary BOC-CN-Bi heterojunction composites still possess high photocatalytic performance for RhB degradation compared to the reported BOC-CN photocatalysts. Therefore, we can reach the conclusion that metal Bi has a remarkable effect on enhancing photocatalytic performance. To explore the kinetic reaction, the kinetics of RhB degradation for all samples are analyzed. The degradation data of all catalysts are linearly fitted according to the equation:

$$\mathrm{Ln}\left(\frac{C}{C_{0}}\right)=\mathrm{kt}+a$$

where C, C_0_ and k are the concentration of RhB under the different times, the concentration of RhB after the adsorption-desorption equilibrium, and the apparent reaction rate constant, respectively. As shown in Figure 6B, the relationship between Ln(C/C_0_) and t of all samples conforms to the first-order kinetic model. The k of every sample is estimated and shown (Figure 6B). Obviously, BOC-CN-Bi possesses the fastest photodegradation rate, which is 6.2 and 3.5 times than that of BOC and CN. The aforementioned results forcefully verified that BOC-CN-Bi ternary composites possess a superior photocatalytic activity. We also explored the effects of pH, dye concentration and catalyst amount on photocatalytic degradation (Figure S2). Obviously, the more photocatalytic content, the better the degradation effect. However, the degradation efficiency and the catalyst increase disproportionately. Moreover, the concentration of organic pollutants increased, and the degradation efficiency decreased slightly. When pH increased, the degradation efficiency decreased significantly. When pH decreased, the catalyst completely adsorbed organic pollutants after darkness, and the degradation efficiency could not be measured (Figure S3, Supplementary Materials). It is well known that the stability of photocatalysts is crucial in practical applications. Therefore, the cycling experiments of BOC-CN-Bi composites were carried out. As shown in Figure 6C, the photocatalytic performance is not significantly reduced in three cycles, which confirms the outstanding stability of the composites.

Under photocatalytic degradation, organic pollutants are decomposed by active species, e.g., active holes (h^+^), hydroxyl radicals (•OH) and superoxide radicals (•O_2_^−^). To verify the dominant active species of BOC-CN-Bi ternary composites under photocatalytic degradation, trapping experiments are performed by adding different scavengers (Figure 6D). In the experiment, benzoquinone (BQ), sodium oxalate (Na_2_C_2_O_4_) and isopropanol (IPA) are used as a quencher of •O_2_^−^, h^+^ and •OH. After adding IPA, the degradation efficiency decreases slightly, implying that •OH is not decisive active species in the photodegradation experiment. Evidently, when BQ and Na_2_C_2_O_4_ are added, the degradation efficiency of pollutants is remarkably suppressed, which could declare that •O_2_^−^ and h^+^ species have a pivotal role in degrading pollutants. More importantly, the degradation rate of pollutants for Na_2_C_2_O_4_ is obviously lower than that of BQ, suggesting that h^+^ is a more vivacious species. The results evidently demonstrate that •O_2_^−^ and h^+^ are the main active species, and h^+^ acts in a dominant role for RhB degradation.

**Figure 7:**
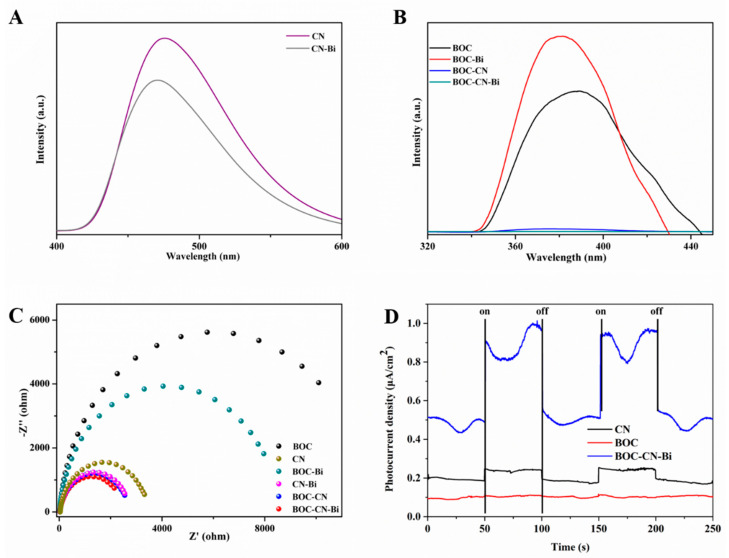
The PL (**A**,**B**), Nyquist (**C**) and photocurrent spectra (**D**) of over as-prepared samples.

Photoluminescence (PL) is used to study the electron-hole separation efficiency. The PL spectra of all samples are shown in Figure 7A,B. Commonly, the lower PL intensity signifies the lower recombination of electron-hole pairs [38,39]. CN-Bi has lower PL intensity compared with pure CN (Figure 7A). As shown in Figure 7B, the PL spectra of BCO-CN and BCO-CN-Bi obviously reduce, suggesting that CN and Bi can restrain the carrier recombination of BCO. Among all samples, BCO-CN-Bi has the lowest PL spectra, indicating that BCO-CN-Bi has the highest carrier separation efficiency. To further study the behaviors of charge transfer for as-prepared samples, the EIS and transient photocurrent responses are carried out. It is well known that the smaller the radius of the arc, the smaller the interfacial resistance [40,41]. As disclosed in Figure 7C, BOC-CN-Bi photocatalysts possess the smallest arc compared to pure BOC, CN and other binary samples, which implies the lowest charge transfer resistance. We also execute photocurrent measurement to further investigate the separation rate of e^−^-h^+^ pairs for samples. The photocurrent intensity of BOC-CN-Bi photocatalysts was much stronger than BOC and CN alone (Figure 7D), explaining that the designed ternary Z-scheme heterojunction among BOC, Bi and CN boosts the separation efficiency of photoinduced carriers [42].

**Figure 8:**
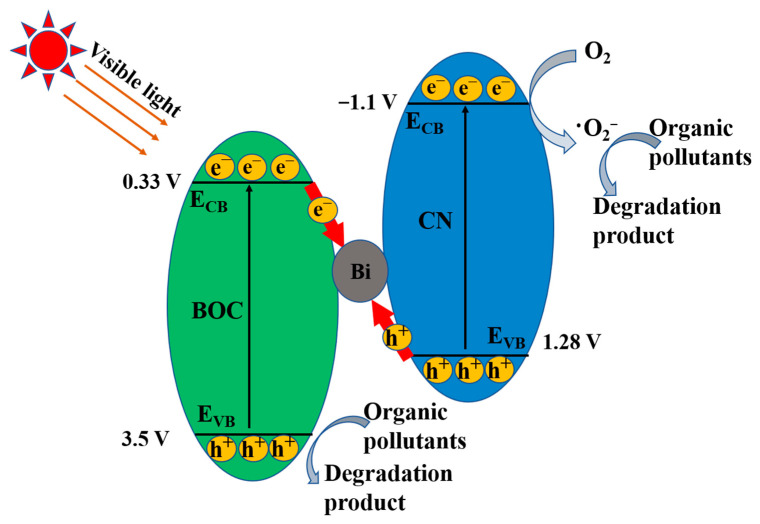
The schematic of BOC-CN-Bi Z-scheme heterojunction by degrading RhB.

Mott-Schottky was used to study the conduction band of CN. The conduction band of CN is −1.1 V. According to our previous studies, BOC has a conduction band of 0.33 V [43]. The valence band of BOC and CN is severally 3.5 and 1.28 V according to E_g_ = E_VB_ − E_CB_. In consideration of the dominant active species, the elevated carrier separation efficiency and the relative band positions of BOC and CN, we designed and discussed a novel indirect heterojunction system to expatiate the feasible reaction mechanism of BOC-CN-Bi photocatalysts for degrading pollutants (Figure 8). When illuminated by visible light, both BOC and CN could produce photoinduced electrons in the conduction band. Simultaneously, the valence band of BOC and CN left a lot of holes. Metal Bi with excellent conductivity could readily capture and transmit electrons. Therefore, the electrons of BOC tended to transfer to metal Bi and consumed the holes of CN because of their different band level, greatly improving the separation efficiency of e^−^–h^+^ pairs. The bi bridge was responsible for the elevated separation efficiency of carriers between BOC and CN, which was proved by the mentioned electrochemical and PL results. As a result, the electrons of BOC combined with the holes of CN resulted in the agglomeration of h^+^ in the VB of BOC (3.5 V) and e^−^ in the CB of CN (−1.1 V) for efficient carrier separation. The reductive electrons in the CB of CN could reduce the absorbed O_2_ to O_2_^−^ due to E(O_2_/O_2_^−^) = −0.33 V [44,45]. Ultimately, the holes of BOC together with the produced ∙O_2_^−^ were able to degrade the adsorbed organic pollutants. The remarkably better photocatalytic degradation performance for the ternary BOC-CN-Bi composites was beneficial from the intensive separation of carriers and the extended visible-light absorption range.

## 3. Materials and Methods

### 3.1. Preparation of Photocatalyst

The preparation of g-C_3_N_4_: firstly, the melamine was thoroughly ground. The obtained powders were then calcined at 550 °C (heating rate: 5 °C min^−1^) for 3 h in a muffle furnace. Until they reached room temperature, the products were ground for further use and labeled as CN.

The preparation of Bi_2_O_2_CO_3_ and g-C_3_N_4_-Bi_2_O_2_CO_3_: 0.238 g g-C_3_N_4_, 0.5 g cetyl trimethyl ammonium bromide and 2.99 g Na_2_CO_3_ were added into 40 mL deionized water under continuous stirring, and this was labeled as solution A. Meanwhile, 1.71 g Bi(NO_3_)_3_·5H_2_O and 10 mL HNO_3_ (1 mol L^−1^) were added into 40 mL deionized water under continuous stirring to form solution B. We then mixed solution A and B and continued stirring for 3 hours. After that, the products were obtained via centrifugation, washed and dried, labeling them as BOC-CN. Without adding CN, Bi_2_O_2_CO_3_ (BOC) was synthesized.

The preparation of g-C_3_N_4_-Bi, Bi/Bi_2_O_2_CO_3_ and g-C_3_N_4_@Bi/Bi_2_O_2_CO_3_: 1.71 g Bi(NO_3_)_3_·5H_2_O was added into ethylene glycol (EG). After stirring for 2 h, 0.238 g g-C_3_N_4_ was added to the above solution and was stirred for 3 h. Finally, the mixed solution was heated at 180 °C for 10 h in a reaction kettle. The products were obtained via centrifugation, washed and dried, and labeled as CN-Bi. Similarly, Bi/Bi_2_O_2_CO_3_ and g-C_3_N_4_@Bi/Bi_2_O_2_CO_3_ were prepared by replacing 0.238 g g-C_3_N_4_ with 0.238 g Bi_2_O_2_CO_3_ or g-C_3_N_4_-Bi_2_O_2_CO_3_ during synthesis, and labeled as BCO-Bi and BOC-CN-Bi, respectively.

### 3.2. Materials Characterization

The elementary composition, phase structure and morphology of samples were measured by X-ray photoelectron spectroscopy (XPS), X-ray diffraction (XRD), scanning electron microscopy (SEM), and transmission electron microscopy (TEM), respectively. The UV-vis diffuse reflectance spectra (DRS) and photoluminescence (PL) of samples were obtained by a Lambda 950 Spectrophotometer and Fluorescence Spectrophotometer. The electrochemical impedance spectroscopy (EIS) and photocurrent response were recorded via an electrochemical workstation (CHI660E). The detailed condition of the electrochemical tests is the same as we reported before.

### 3.3. Photocatalytic Experiments

The degradation of RhB dyes was utilized to determine the photocatalytic degradation performance of all samples via a 400 W Xe lamp (≥420 nm). Before each experiment, 30 mg samples were adequately dispersed in a 30 mL RhB solution. In the dark, the obtained suspension solution was consumingly stirred for 1 h on behalf of an adsorption-desorption equilibrium. During the photoreaction process, 3 mL solution was collected every 30 minutes and centrifuged. A UV-vis spectrophotometer was used to test the RhB concentration in the culture at 553 nm.

## 4. Conclusions

To sum up, we have successfully fabricated a novel BOC-CN-Bi Z-scheme heterojunction. As shown in UV-vis DRS, the BOC-CN-Bi composites have a wider visible-light absorption range than pure BOC. Bi metal as an electron bridge significantly improves the separation efficiency of photogenerated carriers in a heterojunction composite, which is demonstrated by the photocurrent and PL. The BOC-CN-Bi composites possess significantly superior photocatalytic degradation activity compared to single BOC and CN. Radical trapping studies identify that h^+^ and •O_2_^−^ are two primary active species during the degradation reaction. Cycling tests manifest the remarkable stability of the BOC-CN-Bi heterojunction composites. This work implies that BOC-CN-Bi photocatalysts might be efficient and promising novel materials for removing organic pollutants from wastewater.

## Data Availability

Not applicable.

## Supplementary Materials

The following supporting information can be downloaded at: https://www.mdpi.com/article/10.3390/molecules27238336/s1, Figure S1: The band gaps of BOC-Bi, CN-Bi, BOC-CN, and BOC-CN-Bi; Figure S2: The photodegradation different organic pollutant of BOC-CN-Bi under different conditions; Figure S3: The color of the solution for BOC-CN-Bi after the dark at pH = 4; Figure S4: Mott-Schottky plots of CN; Table S1: Element content of BOC-CN-Bi composites by EDS.
