# Supplementary material for: Fabrication of Novel g-C3N4@Bi/Bi2O2CO3 Z-Scheme Heterojunction with Meliorated Light Absorption and Efficient Charge Separation for Superior Photocatalytic Performance
Source: Molecules. 2022 Nov 29;27(23):8336. doi: 10.3390/molecules27238336 (PMC9740476; doi:10.3390/molecules27238336)
Supplement: Supplementary file 1 [file molecules-27-08336-s001.zip › molecules-2051206-supplementary-done.pdf]

molecules

MDPI

Supplementary Materials

Fabrication of Novel g-C_3N_4@Bi/$Bi_2O_2CO_3$ Z-Scheme Heterojunction with Meliorated Light Absorption and Efficient Charge Separation for Superior Photocatalytic Performance

Hongxia Fan, Xiaohui Ma, Xinyang Li, Li Yang, Yongzhong Bian*, Wenjun Li*

Beijing Key Laboratory for Science and Application of Functional Molecular and Crystalline Materials, University of Science and Technology Beijing, Beijing 100083, China

Figures

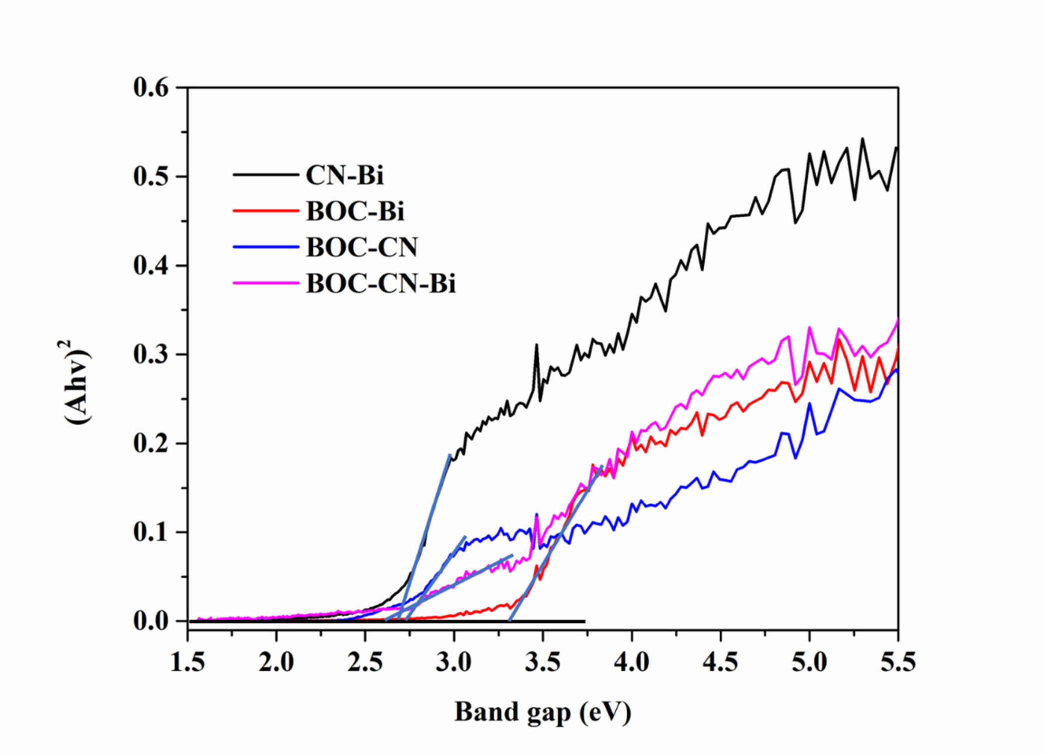

Figure S1. The band gaps of BOC-Bi, CN-Bi, BOC-CN, and BOC-CN-Bi.

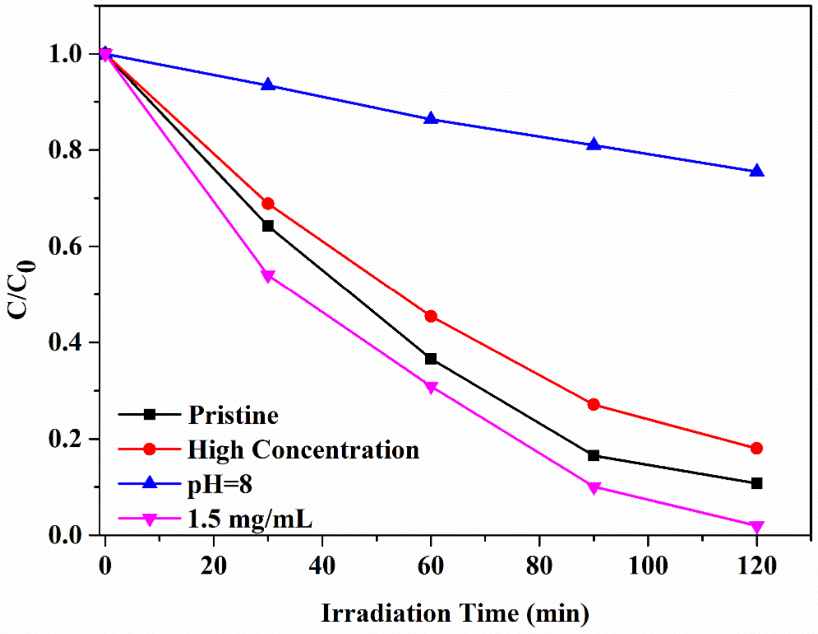

Figure S2. The photodegradation different organic pollutant of BOC-CN-Bi under different conditions.

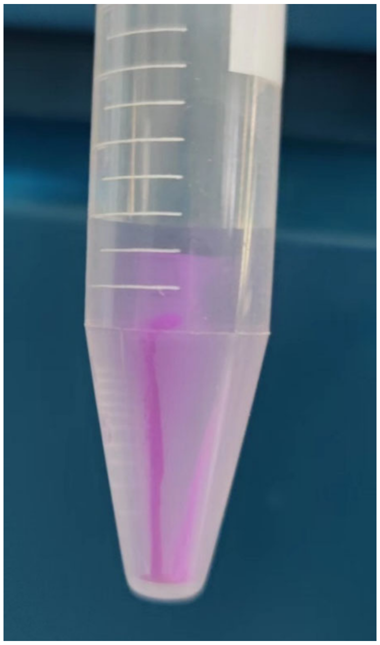

Figure S3. The color of the solution for BOC-CN-Bi after the dark at pH = 4.

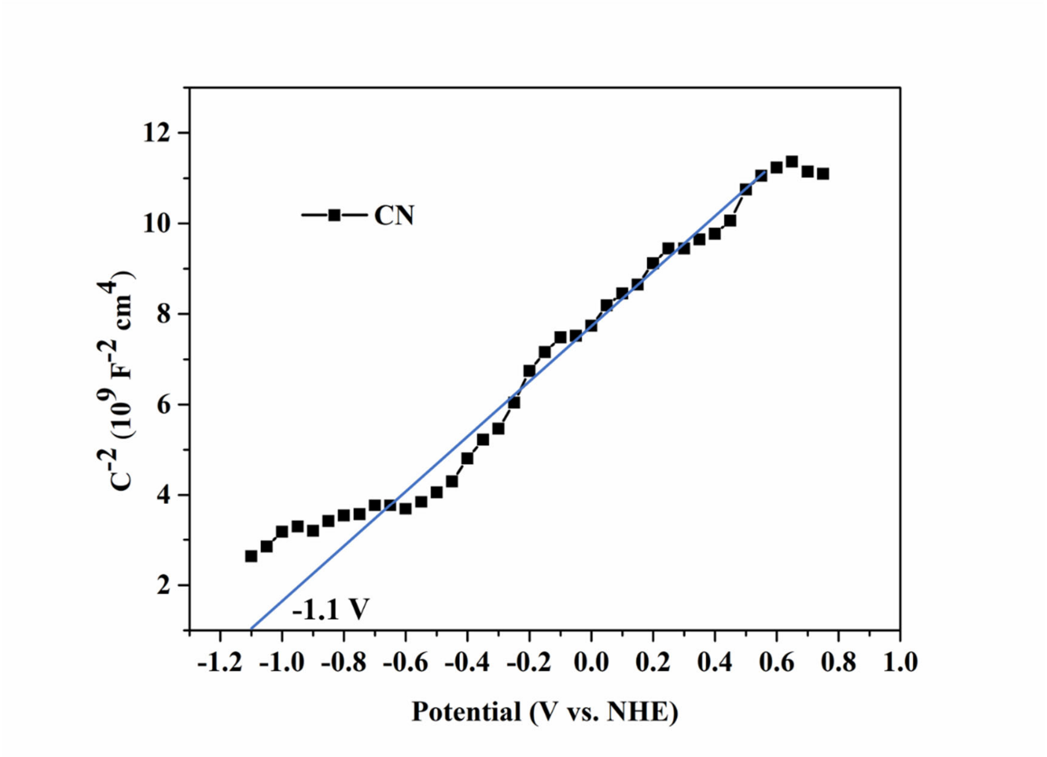

Figure S4. Mott-Schottky plots of CN.

Table S1. Element content of BOC-CN-Bi composites by EDS.

Element	Weight percentage	Wt % Sigma	Atomic percent
C	16.11	0.58	46.86
N	1.41	0.72	3.53
O	17.76	0.42	38.79
Bi	64.72	0.78	10.82

Table S2. The Value of RhB adsorption for all samples.

	BOC	CN	BOC-Bi	CN-Bi	BOC-CN	BOC-CN-Bi
Value of RhB adsorption	0.213	0.181	0.225	0.193	0.211	0.256
